# Supplementary material for: Scalable miniature on-chip Fourier transform spectrometer for Raman spectroscopy
Source: Light Sci Appl. 2025 May 30;14:208. doi: 10.1038/s41377-025-01861-7 (PMC12122837; doi:10.1038/s41377-025-01861-7)
Supplement: Supplementary file 1 — Supplementary Information for Scalable Miniature On-chip Fourier Transform Spectrometer For Raman Spectroscopy [file 41377_2025_1861_MOESM1_ESM.docx]

Supplementary Information for

Scalable Miniature On-chip Fourier Transform Spectrometer For Raman Spectroscopy

*Sarp Kerman^1,2,*^, Xiao Luo^2^, Zuoqin Ding^2^, Zhewei Zhang^3^, Zhuo Deng^3,†^, Xiaofei Qin^2^, Yuran Xu^2,4^, Shuhua Zhai^2,‡^, Chang Chen^1,2,5,*^*

^1^Institute of Medical Chip, Ruijin Hospital, Shanghai Jiao Tong University School of Medicine, 200025, Shanghai, China

^2^Shanghai Photonic View Technology Co., Ltd., 200443, Shanghai, China.

^3^Shanghai Industrial μTechnology Research Institute (SITRI), 201800, Shanghai, China.

^4^Friedrich Schiller University Jena, 07743, Jena, Germany

^5^State Key Laboratory of Transducer Technology, Shanghai Institute of Microsystem and Information Technology, Chinese Academy of Sciences, Shanghai, 200050, China.

†Present Address: Advanced Biomedical Instrumentation Centre, Hong Kong, Hong Kong SAR, China.

‡Present Address: Energy Singularity Energy Technology Shanghai Co. Ltd., Shanghai, China.

^*^Corresponding Authors

e-mail: [sarp.kerman@photonicview.com](mailto:sarp.kerman@photonicview.com), [changchen@sjtu.edu.cn](mailto:changchen@sjtu.edu.cn)

Table of Contents

[S1. Fourier Transform Spectrometer Design 2](#_Toc194068533)

[Interferometer Design 2](#_Toc194068534)

[Supplementary Fig. S1| Interferometer Structure and Output Response. 5](#_Toc194068535)

[Supplementary Fig. S2| FDTD simulation results showing the variation in output power and interference contrast based on VGC parameters. 7](#_Toc194068536)

[FTS Design 8](#_Toc194068537)

[Supplementary Fig. S3| Layout of the interferometer. 8](#_Toc194068538)

[S2. Optical Throughput 8](#_Toc194068539)

[Single Mode Waveguide Optical Throughput 8](#_Toc194068540)

[Optical Throughput of the Fourier Transform Spectrometer 9](#_Toc194068541)

[S3. Fabrication Verification, Interferometer Efficiency Simulation and Component Measurements 9](#_Toc194068542)

[Fabrication Verification 9](#_Toc194068543)

[Supplementary Fig. S4| Cross-section scanning electron microscope (SEM) images and top-view bright field images of the chip. 12](#_Toc194068544)

[Interferometer Efficiency Simulation 12](#_Toc194068545)

[Supplementary Fig. S5| Simulated output power efficiency of an interferometer based on the SEM and T-matrix characterization. 12](#_Toc194068546)

[Component Measurements 13](#_Toc194068547)

[Supplementary Fig. S6| Component measurement results. 14](#_Toc194068548)

[S4. Spectrum Reconstruction 15](#_Toc194068549)

[Measurement Setups and Beam Characterization 15](#_Toc194068550)

[Supplementary Fig. S7| T-matrix measurement and Raman Spectrum reconstruction setups and beam characterization. 17](#_Toc194068551)

[Supplementary Table 1| List of components used for T-matrix measurement and Raman spectrum reconstruction, as illustrated in Supplementary Figures S7a and S7d. 17](#_Toc194068552)

[Interferometer Array Cavity Response in the Raman Spectrum Reconstruction Measurements 18](#_Toc194068553)

[Supplementary Fig. S8| The Raman spectrum interferometer responses for different cavity lengths in the FTS. 18](#_Toc194068554)

[Influence of Signal-to-Noise Ratio (SNR) on Spectrum Reconstruction for Different Regression Methods 19](#_Toc194068555)

[Supplementary Fig. S9| Spectrum reconstruction using different regression methods with added noise to the cavity response. 22](#_Toc194068556)

[Measured Raman Spectrum Reconstruction Dependence On The Regression Method 22](#_Toc194068557)

[Supplementary Fig. S10| Raman spectra of four substances reconstructed from the measurements using different regression methods. 23](#_Toc194068558)

[Measured Raman Spectrum Reconstruction Dependence On The Number of Images 23](#_Toc194068559)

[Supplementary Fig. S11| Raman spectra of four substances reconstructed from the measurements using varying numbers of images and LASSO regression. 24](#_Toc194068560)

[Measured Raman Spectrum Reconstruction Using LASSO Regression With Varying Hyperparameter Value 25](#_Toc194068561)

[Supplementary Fig. S12| Raman spectra of four substances reconstructed from the measurements using varying LASSO regression hyperparameter values. 25](#_Toc194068562)

[S5. Concentration Detection From The Reconstructed Spectra 26](#_Toc194068563)

[Supplementary Fig. S13| Concentration detection of IPA in water solution using the peak amplitude of its Raman spectra reconstructed from the FTS. 27](#_Toc194068564)

[S6. Total Efficiency of the Waveguide-Based FTS Raman Spectroscopy Setup 28](#_Toc194068565)

[Supplementary Table 2| The breakdown of the losses of waveguide-based FTS Raman setup. 29](#_Toc194068566)

[Supplementary References 29](#_Toc194068567)

# S1. Fourier Transform Spectrometer Design

## Interferometer Design

In this section, we detail the design principle of our interferometer using a numerical model. The interferometer features a vertical grating coupler (VGC) connected to an $8 \mu m$ wide waveguide, which is terminated by an Al mirror. Supplementary Figure S1a provides a schematic representation of the interferometer’s physical structure, including its input and output ports. To better understand its operation and predict the wavelength-dependent output power response, we developed a numerical model based on the propagation of the E-field. This model employs 3x3 scattering matrices (S-matrices) for each component, the 3^rd^ port representing the power lost from the system^1^. Each S-matrix was constructed with port 1 on the left side, port 2 on the right side, and port 3 representing the scattered power. The following expression was iteratively calculated 20 times to ensure saturation of the result, and the output E-field of all ports for each component was stored. Each iteration involved multiplying the expression below, from right to left, and assigning the E-field between ports 1 and 2 of the neighboring components.

$S_{m}\leftrightarrow S_{c}\leftrightarrow S_{g5}\leftrightarrow S_{g4}\leftrightarrow S_{g3}\leftrightarrow S_{g2}\leftrightarrow S_{g1}\leftarrow E_{in}$ (1)

Where $E_{in}$ represents the input E-field of the interferometer, and $S_{gn}$ , $S_{c}$ and $S_{m}$ are the S-matrices of the n-th VGC grating period, the cavity, and the mirror, respectively. These terms are expressed below.

$E_{in}= \left[ \begin{matrix} 1 \\ 0 \\ 0 \end{matrix} \right]$ (2)

$S_{g,n}= \left[ \begin{matrix} \left| b \right|.e^{i\pi} & \left| a \right|.e^{-ikpn_{gr}} & 0 \\ \left| a \right|.e^{-ikpn_{gr}} & \left| b \right|.e^{i\pi} & 0 \\ \sqrt{1-\left| a \right|^{2}-\left| b \right|^{2}} & \sqrt{1-\left| a \right|^{2}-\left| b \right|^{2}} & 0 \end{matrix} \right]$ (3)

$S_{c}= \left[ \begin{matrix} 0 & e^{-ik(L-\frac{p-\boldsymbol{e}}{2})n_{eff}} & 0 \\ e^{-ik(L-\frac{p-\boldsymbol{e}}{2})n_{eff}} & 0 & 0 \\ 0 & 0 & 0 \end{matrix} \right]$ (4)

$S_{m}= \left[ \begin{matrix} \sqrt{R}.e^{i\pi} & 0 & 0 \\ 0 & \sqrt{R}.e^{i\pi} & 0 \\ 0 & 0 & 0 \end{matrix} \right]$ (5)

Where $a$ and $b$ are the transmission and reflection coefficients of a grating line (from FDTD simulations), $k=2\pi/\lambda$ is the amplitude of the wave vector in vacuum, $p$ and $\boldsymbol{e}$ are the pitch and the etch width of the VGC, $L$ is the cavity length, $n_{eff}\approx1.67$ is the effective refractive index of the $8 \mu m$ wide cavity, $n_{gr}=\left( \frac{n_{eff}.\left( p-\boldsymbol{e} \right)+n_{SiO_{2}}.\boldsymbol{e}}{p} \right)$ is the estimated effective refractive index inside the VGC, and $R=0.9$ is the reflectivity of the mirror.

We accounted for the modulation of transmission and reflection in each grating line depending on scattering efficiency at different wavelengths. This was achieved by scaling the E-field amplitudes in ports 1 and 2 based on the amplitude in port 3. The intensity inserted into each grating line was calculated as:

$I_{in}=\left| E_{in,1} \right|^{2}+\left| E_{in,2} \right|^{2}$ (6)

Where $E_{in,1}$ and $E_{in,2}$ are the input E-fields from port 1 and port 2, respectively. After accounting for scattered power, the remaining intensity was used to rescale the output fields of ports 1 and 2:

${E_{out,1,2}}^{\dagger}= E_{out,1,2}.\frac{I_{in}-\left| E_{out,3} \right|^{2}}{\left| E_{out,1} \right|^{2}+\left| E_{out,2} \right|^{2}}$ (7)

Supplementary Figure S1b shows the wavelength-dependent amplitude of the output E-field at port 3 (scattering port) for each grating line, using design parameters for the shortest cavity ($L=4.555 \mu m$). The profile demonstrated optimal interference contrast for individual grating lines, with responses converging near the Bragg reflection wavelength (~870 nm) but clearly shifting at other wavelengths. This factor decreases the overall interference contrast as previously discussed.

We computed the cumulative output response of the VGC by summing the output fields from individual grating lines ($E_{out,n}(\lambda)$) and factoring in the phase difference for each angle in the far-field, within $\pm8^{^{\circ}}$, corresponding to the $NA=0.14$ of our optical system.

$E_{out,VGC}\left( \lambda,\theta\right)=\sum_{n=0}^{4} E_{out}\left( n,\lambda\right).e^{ik.np\sin\theta.n_{SiO2}}$ (8)

Supplementary Figure S1c shows the far-field profile’s wavelength variation as derived from this equation.

In some cases, such as comparison with measurement results, we included the effects of the VGC reflector and the top-oxide-air interface. For the VGC reflector, half of the resulting E-field (by intensity) was reflected by the metal layer and recombined with the other half after a phase shift:

$E_{out,VGC}^{w. metal}\left( \lambda,\theta\right)=\frac{E_{out,VGC}\left( \lambda,\theta\right)}{\sqrt{2}}\left( 1+\sqrt{R}e^{ik.2\left( h+\frac{t_{SiN}}{2} \right)\left( \frac{n_{SiO2}}{\cos\theta}-n_{gr}\tan\theta\right)+i\pi} \right)$ (9)

Where $h$ is the distance between the reflector and the waveguide, and $t_{SiN}$ is the thickness of the waveguide layer.

When the top-oxide-air interface was considered, the Fresnel transmission ($t_{s}$) and reflection ($r_{s}$) coefficients were computed as follows:

$r_{s}=\frac{n_{SiO2}\cos\theta-\cos\theta_{air}}{n_{SiO2}\cos\theta+\cos\theta_{air}}$ (10)

$t_{s}=\frac{2n_{SiO2}\cos\theta}{n_{SiO2}\cos\theta+\cos\theta_{air}}$ (11)

Where $\theta_{air}$ is the angle in air calculated via Snell’s law. When there was no reflector, the output E-field took the form:

$E_{out,VGC}^{w. TOX}\left( \lambda,\theta\right)= E_{out,VGC}\left( \lambda,\theta\right).t_{s}$ (12)

When both the reflector and top-oxide-air interface were considered, we included multiple reflections between them:

$E_{out,VGC}^{w. TOX+metal}\left( \lambda,\theta\right)= \sum_{m=0}^{\infty} E_{out,VGC}\left( \lambda,\theta\right).\left( r_{s}\sqrt{R}e^{ik.2\left( h+t_{TOX}+t_{SiN} \right)\left( \frac{n_{SiO2}}{\cos\theta}-n_{gr}\tan\theta\right)+i\pi} \right)^{m}$ (13)

This summation was iterated three times due to computational limits while ensuring the results' saturation. The final output signal was calculated as:

$I_{out,VGC}\left( \lambda\right)= \sum_{\theta} \left| E_{out,VGC}\left( \lambda,\theta\right) \right|^{2}$ (14)

For comparison with FDTD simulations (due to the calculation of the transmission in the software), the output signal was calculated as:

$I_{out,VGC}\left( \lambda\right)= \sum_{\theta} \left| E_{out,VGC}\left( \lambda,\theta\right) \right|^{2}\cos\theta$ (15)

Several assumptions were made in this model and potential sources of variation include: (1) grating lines were treated as point sources, though they may exhibit angular directionality and broader peaks/dips due to interference of the scattered wave along them; (2) the effective refractive index of the grating was averaged; (3) the reflection phase from the grating lines may differ slightly from π; (4) the spectral response of metal reflections was simplified; (5) we did not account for transmission, diffraction, and recoupling of reflected light from metal and TOX ; (6) Supplementary Equation 13 assumes the first reflection from the reflector is in phase with the outgoing light from the VGC.

The model used the parameters for a waveguide thickness of $t_{SiN}=150 \mathrm{nm}$ and a mirror inclination angle of $0^{^{\circ}}$ for the pre-fabrication analysis such as Supplementary Figures S1 and S2. In order to compare the model with the measurement results, post-fabrication variations were considered as $t_{SiN}=136 \mathrm{nm}$ and a mirror inclination angle of $6^{^{\circ}}$.


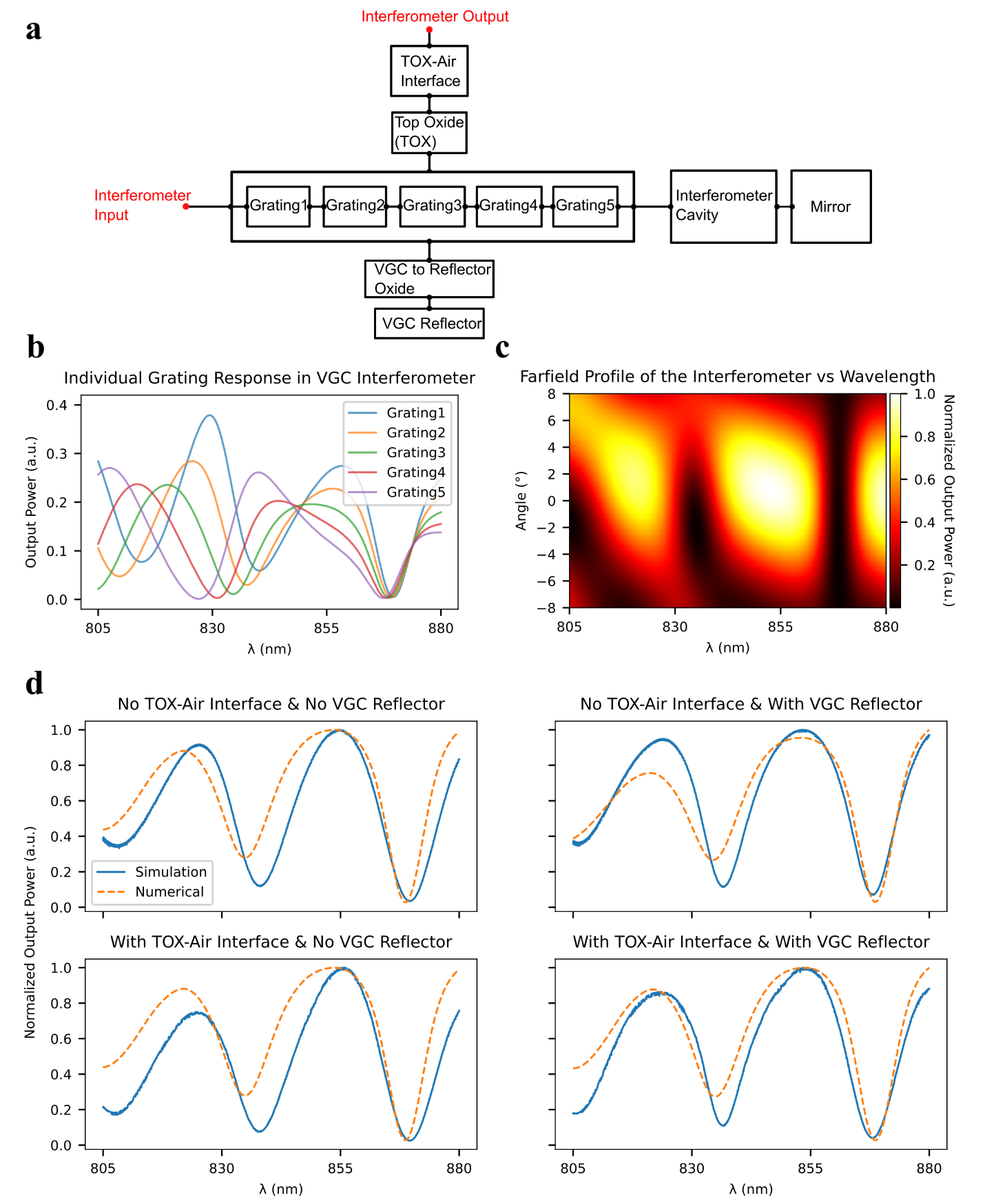


Supplementary Fig. S1| Interferometer Structure and Output Response. **a**, Schematic representation of the interferometer structure, showing the vertical grating coupler (VGC) connected to an $8 \mu m$ wide waveguide and terminated by an Al mirror. **b**, Output response of individual grating lines in the VGC for an interferometer with a cavity length of $L=4.555 \mu m$ , as obtained from the numerical model. **c**, Spectral far-field profile of the interferometer derived from the numerical model, showing the wavelength-dependent variation. **d**, Comparison of the numerical model’s spectral output response with FDTD simulations for four different cases.

Supplementary Figure S1d compared the spectral output response from the VGC under four different conditions: (1) without a top-oxide-air interface or reflector, (2) with only the reflector, (3) with only the top-oxide-air interface, and (4) with both the top-oxide-air interface and reflector. Our numerical model closely aligns with the simulation results, particularly near the Bragg reflection wavelength (870 nm), validating the model’s accuracy. However, deviations in peak positions and interference contrast at shorter wavelengths suggest that phase variations between the grating lines may differ from initial assumptions, possibly due to the simplifications of point source scatterers and the effective refractive index in the grating.

Supplementary Figure S2 presents the FDTD simulation results for the same interferometer cavity length, exploring variations in VGC parameters. Figure S2a illustrates the spectral output response of the VGC for different pitches and etch widths. Figures S2b and S2c show the maximum output power and the interference contrast derived from Figure S2a. Based on these results, we optimized three key aspects during the design process: (1) maximizing output power, (2) maximizing interference contrast, and (3) keeping the critical dimension around 250 nm to minimize fabrication variability. Ultimately, we chose a 567 nm pitch and249 nm etch width, although several other combinations provided comparable results.

Supplementary Figures S2d and S2e demonstrate how the output power and interference contrast vary with the number of grating lines in the VGC. As predicted by our model, increasing the number of grating lines leads to an initial rise in the output power, which eventually saturates, while the interference contrast decreases. After five grating lines, the output power increase becomes insignificant, while the interference contrast rapidly decreases. Based on this, we decided to use five grating lines in the VGC.
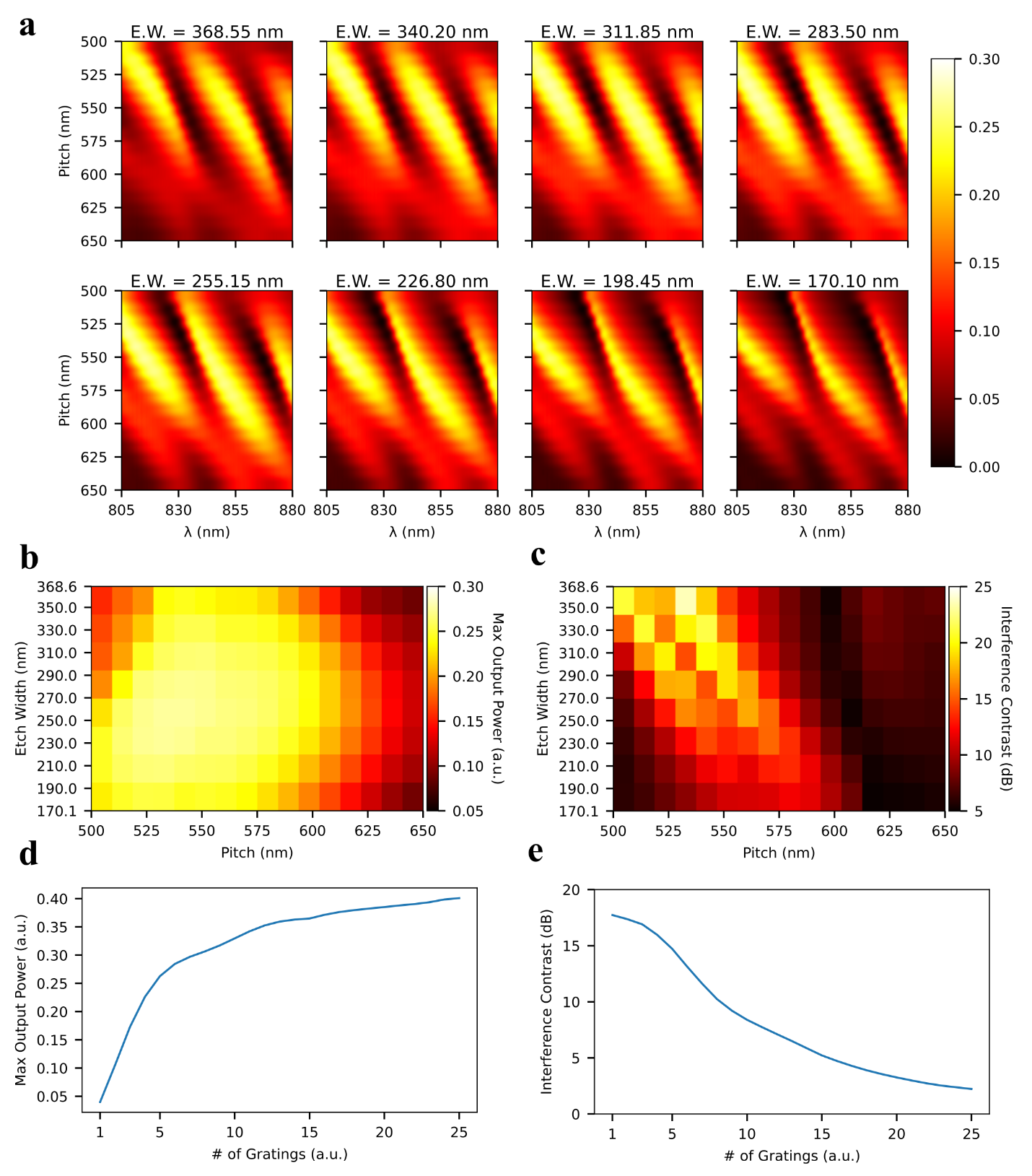


Supplementary Fig. S2| FDTD simulation results showing the variation in output power and interference contrast based on VGC parameters. **a**, Spectral output power as a function of varying etch width and pitch of the VGC. **b**, Maximum output power variation with respect to the etch width and the pitch. **c**, Interference contrast variation for different etch widths and pitches of the VGC. **d**, Maximum output power for varying number of grating lines in the VGC. **e**, Interference contrast as a function of the number of grating lines in the VGC.

## FTS Design


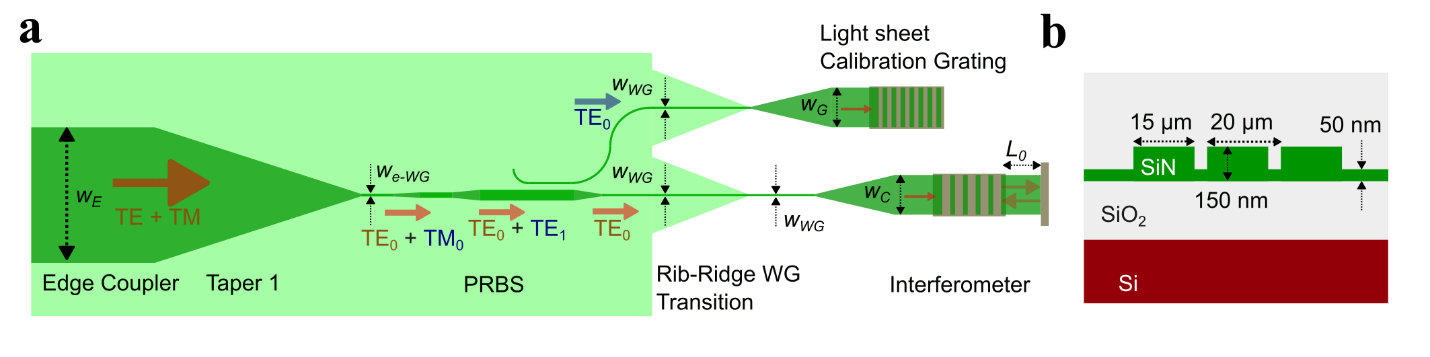


Supplementary Fig. S3| Layout of the interferometer. **a**, Complete layout of the interferometer, where light green indicates the 50 nm slab thickness. **b**, Cross-section of the edge coupler array.

In this section, we provide further details on the FTS design and layout. Supplementary Figure S3a illustrates the layout of the structure. We employed an array of edge couplers with a slab thickness of 50 nm, a width of 15 µm, and a 20 µm pitch, as shown in Supplementary Figure S3b. After the edge coupler, the waveguide width was tapered to a single-mode condition ($w_{e-WG}=600 \mathrm{nm}$) within this stack. To facilitate the calibration of the input power using the coupled TM mode, we incorporated a polarization-rotating beam splitter (PRBS). The PRBS converts the TM_0_ mode to TE_0_ and directs it to the calibration output, while the TE_0_ mode continues propagation, nearly unaffected, to the FTS.

# S2. Optical Throughput

## Single Mode Waveguide Optical Throughput

In this section, we derive the optical throughput of a single-mode waveguide. The TE_0_ mode profile can be approximated as a Gaussian beam^3^. Therefore, we begin our derivation using Gaussian optics equations. The optical throughput $G$ of a source is given by^4^:

$G= \pi S{NA}^{2}$ (16)

where $S$ is the area and $NA$ is the numerical aperture of the beam. The mode field diameter (MFD) of a Gaussian beam at its focus is:

$MFD= \frac{2\lambda}{\pi NA}$ (17)

Assuming that the optical flux is uniformly distributed over a circle with a diameter equal to the MFD, we can express the area $S$ as:

$S\approx\pi\left( \frac{\lambda}{\pi NA} \right)^{2}$ (18)

Substituting this into the expression for throughput:

$G\approx\lambda^{2}$ (19)

The relation^5^ shows that the optical throughput of a single-mode waveguide is determined solely by the wavelength and is independent of its geometry.

## Optical Throughput of the Fourier Transform Spectrometer

Although light is collected from a large area by each edge coupler, the optical throughput of each interferometer is constrained by the throughput of a single-mode waveguide. This is because higher-order modes are filtered out through tapers in the FTS layout. Our FTS consists of 160 interferometers, which result in an optical throughput of $G=160\lambda^{2}=0.0001 \mathrm{mm}^{2}-sr$ . This throughput is two to three orders of magnitude smaller than that of a typical commercial Raman spectrometer^6^, highlighting a clear roadmap for increasing the number of input apertures in future designs.

The optical throughput of our FTS is limited to a single axis, as all edge couplers are aligned in parallel. Consequently, the throughput is asymmetrical and constrained in the perpendicular axis to the array. To estimate the potential light collection at the lens focus, we consider that the lens has a numerical aperture $NA = 0.55$. The 2D throughput of the system is given by $G= \pi S{NA}^{2}$. Assuming a uniform and circular spot, the throughput can be approximated as a square with side length $a=\pi rNA$, where $r$ is the radius of the spot. For a single-mode waveguide, this simplifies to $a=\lambda$. Thus, the spot size collected into the FTS is limited to $78.7 \mu m$ by $0.5 \mu m$.

For a multi-mode fiber with $105 \mu m$ core diameter and $NA = 0.22$, the collection corresponds to 43 single-mode waveguides on each axis. Since the optical throughput of the FTS is larger than the fiber in one axis, and there is a 43x penalty in the perpendicular axis, the FTS would collect about 2% of the light from the fiber. This limitation underscores the need for a 2D input arrangement in future iterations of our FTS design.

Although the total optical throughput of the FTS presented in this article is 10^2^-10^3^ times smaller than that of a commercial Raman spectrometer^6^, the collection efficiency depends on the scattering area of the signal on the substance. When the optical throughput in the array axis satisfies the signal’s optical throughput, the efficiency is limited only by the single-mode waveguide aperture in the perpendicular axis, making it comparable to a slit of a diffraction-type spectrometer. Therefore, the efficiency difference between these two devices can be approximated by the expression $\pi.s.NA/(2.\lambda)$, where $s$ is the slit width and $NA$ is the numerical aperture of the diffraction-type spectrometer. Consequently, the Raman signal couples into a diffraction-type spectrometer with a 25 µm slit width and f/1.3 17.8 times more efficiently compared to the FTS, making the coupling efficiency 5.6% of a diffraction-type spectrometer.

# S3. Fabrication Verification, Interferometer Efficiency Simulation and Component Measurements

## Fabrication Verification

We verified the stack parameters post-fabrication using a series of SEM images. Supplementary Figures S4a, S4b, S4c, and S4d show cross-sectional SEM images taken from different chip sections. After saw dicing the 200 mm wafer into 22 mm x 22 mm reticles, the reticles were further cleaved to the regions of interest using a diamond pen. The cleaved sections were then dipped in a buffered oxide etch (BOE) solution to enhance the contrast between the SiN and SiO_2_ layers.

Since the cleaving process was performed manually, it may have introduced a small angle at the chip cross-section, resulting in an overestimation of the stack thickness. This verification aimed to confirm the presence of all layers, identify any potential artifacts such as voids, check the width of various structures (which is largely unaffected by the cleaving angle), and provide an estimate of the layer thicknesses.

The complete stack, from the Si substrate to the SiO_2_ coating of the mirror, is shown in Supplementary Figure S4a. We observed voids beneath the Al VGC reflector and above the Al used for the interferometer mirror, but these voids are unlikely to affect performance. The distance from the VGC reflector to the substrate was measured at 4.21 µm, and from the VGC reflector to the top Al mirror was 2.0 µm. The thickness of the VGC reflector and mirror were measured as 0.2 µm and 0.53 µm, respectively.

Supplementary Figure S4b shows a fully etched single-mode waveguide with a sidewall angle of ~83°, a width of 587 nm in the middle (close to the design target of 560 nm), and a thickness of 185 nm (larger than the design target of 150 nm). Supplementary Figure S4c shows a cross-section of a linear grating coupler (LGC) with a design pitch of 630 nm and an etch width of 284 nm. The measured pitch was 630 nm, and an etch width was 278 nm in the middle, both of which were close to the design specifications. The thicknesses of the waveguide and VGC reflector layers were slightly larger than expected, while the distance between the waveguide layer and the reflector matched the design parameter closely.

To accurately verify the waveguide and slab thicknesses, we took a cross-section picture of a PRBS by focused ion beam (FIB) milling. The image, shown in Supplementary Figure S4d, reveals a waveguide thickness of 140 nm and a slab thickness of 45 nm. The image also indicated that the gap (designed vs. measured: 200 nm vs. 260 nm) and waveguide widths at the two ports of the ADC (designed-measured: 1250 nm vs. 1170 nm, and 466 nm vs. 410 nm) deviated significantly from the design parameters.

We captured bright-field images of the top view of the FTS and its components, as shown in Supplementary Figure S4a. A visual inspection was conducted to ensure that there were no visible damages or layout errors after fabrication. Supplementary Figures S4e, S4f, S4g, and S4h present images of the complete FTS structure, the interferometers, the PRBS with the calibration LGC, and the edge coupler sections. After a thorough analysis of these images, we confirmed that no visible deviations from the designed layout were observed.


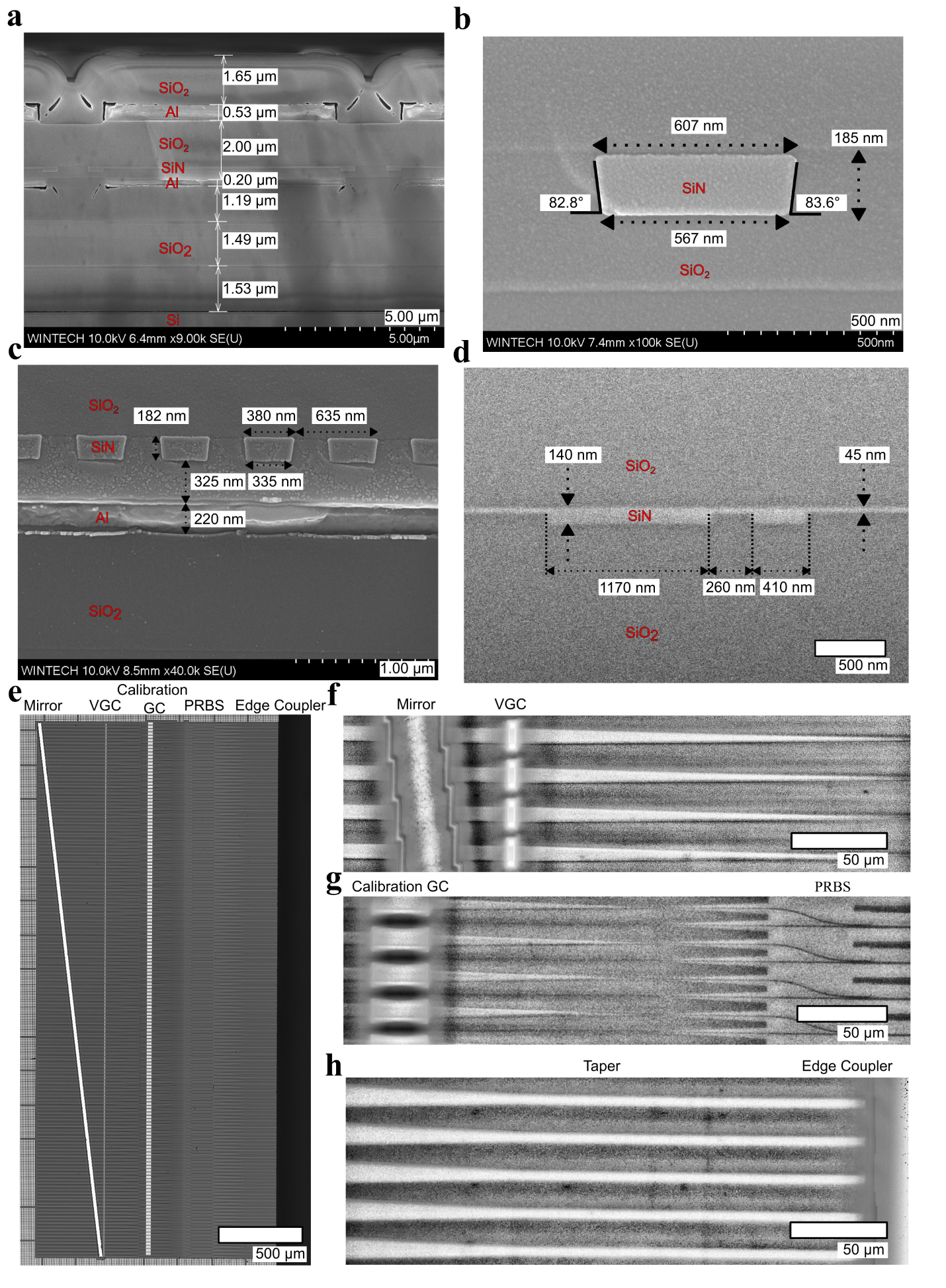


Supplementary Fig. S4| Cross-section scanning electron microscope (SEM) images and top-view bright field images of the chip. **a**, A cross-section SEM image of the entire stack. **b**, A cross-section SEM image of a fully etched single-mode waveguide. **c**, A cross-section SEM image of a linear grating coupler (LGC). **d**, A cross-section image of the adiabatic directional coupler (ADC) section of a polarization rotating beam splitter (PRBS). **e**, A top-view image of the Fourier transform spectrometer (FTS). **f**, A top-view image of four interferometers in an array with varying cavity lengths, where the bright layer represents the waveguides. **g**, A top-view of four PRBS connected to the interferometers and their calibration output ports, where the waveguide appears dark for narrow and bright for wider waveguides. **h**, A top-view image of the edge coupler array, where the waveguide layer appears dark.

## Interferometer Efficiency Simulation


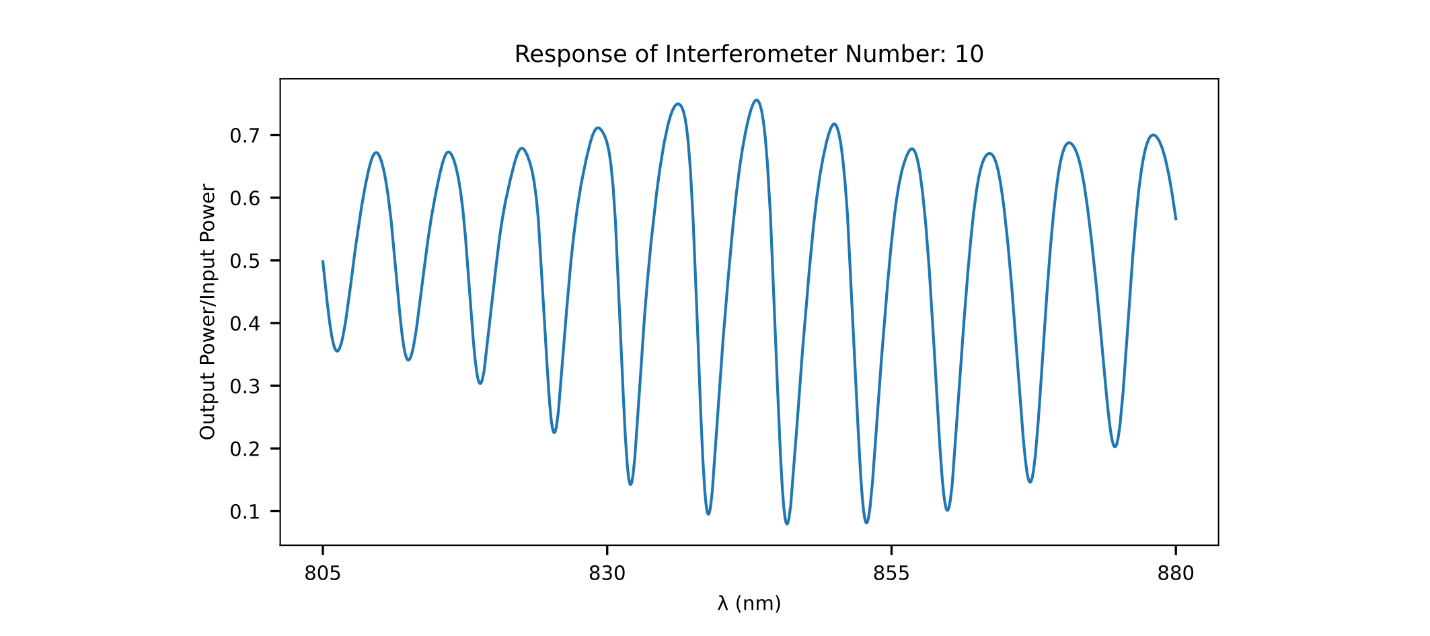


Supplementary Fig. S5| Simulated output power efficiency of an interferometer based on the SEM and T-matrix characterization.

We conducted a 3D FDTD simulation to quantify the output power efficiency of the interferometers and found that up to 75% of the input power, with an average of 49% (~ 3 dB loss), can be directed upward. Supplementary Figure S5 shows the output power efficiency of Interferometer Number 10. The simulation stack and structure were based on SEM characterization and the SiN waveguide thickness (t_SiN_ = 136 nm) which was estimated from T-matrix measurements. The cavity length was selected based on Interferometer Number 10 to ensure a sufficient number of peaks and dips within the spectral range of interest.

## Component Measurements


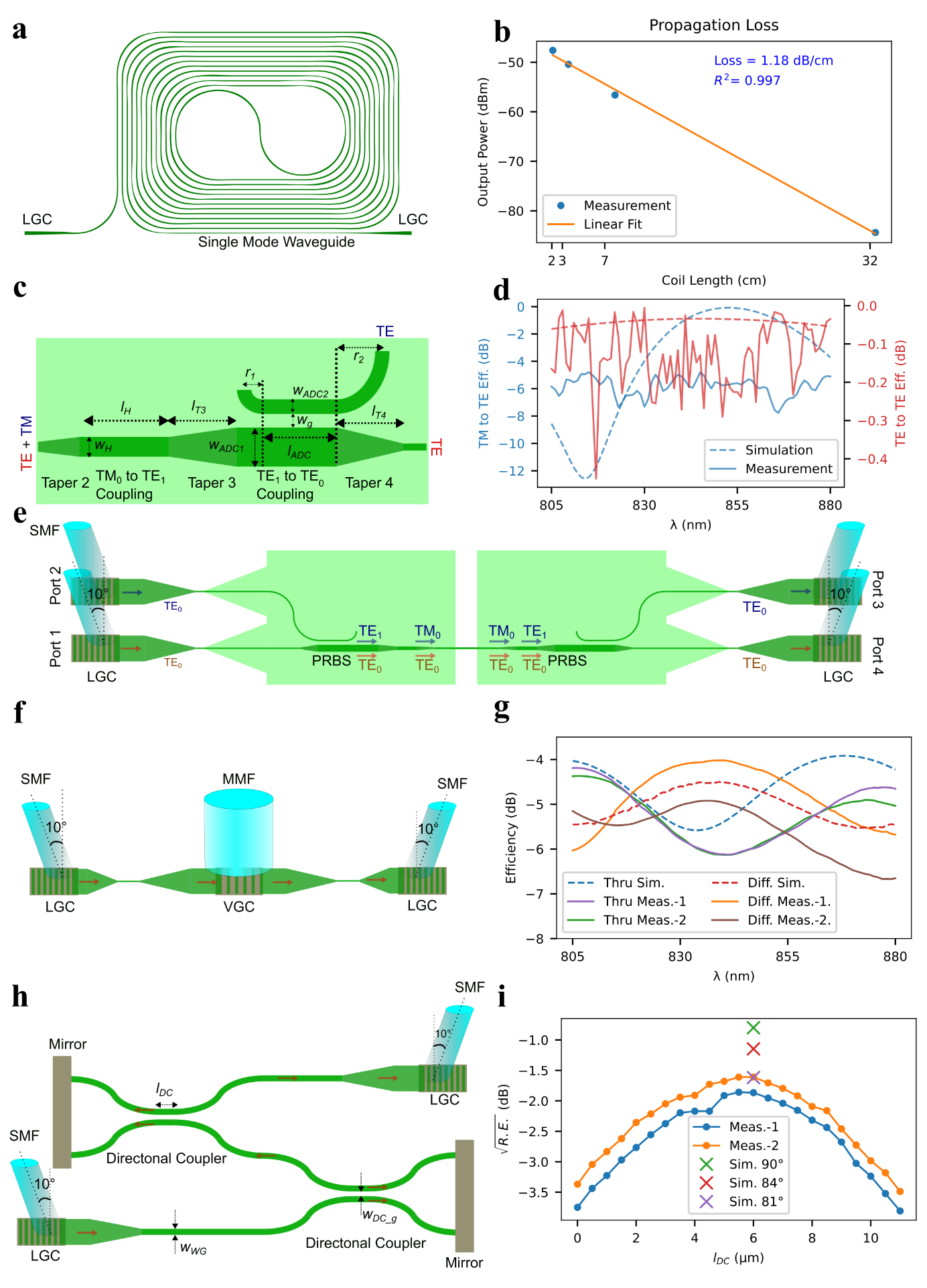


Supplementary Fig. S6| Component measurement results. **a**, Coil structure for single mode waveguide loss measurements. **b**, Output power from the LGC of the coil for varying lengths, and the propagation loss of the single mode waveguide. **c**, Schematic of the polarization rotating beam splitter (PRBS). **d**, Spectral efficiency of the PRBS from both the measurements and simulations. **e**, PRBS chain structure used for efficiency measurement. **f**, Vertical Grating Coupler (VGC) diffraction and transmission measurement structure. **g**, Transmission and diffraction efficiency of the VGC from simulations and measurement results. **h**, Mirror efficiency measurement structure. **i**, Square root of the transmission from the mirror efficiency measurement structure, measured as a function of the directional coupler coupling length.

Supplementary Figure S6 shows the experimental performance of various components discussed throughout this article. The propagation loss of the single-mode waveguide, measured at 1.18 dB cm^-1^ for 860 nm wavelength, was determined using coils with lengths of 2.1 cm, 3.6 cm, 8.0 cm and 32.5 cm, as shown in Supplementary Figure S6a. The output power from each coil, along with the linear fitting used to calculate the propagation loss, is illustrated in Supplementary Figure S6b. Supplementary Figures S6c, S6d, and S6e display the PRBS structure, measurement results, and the measurement structure’s schematic, respectively. The PRBS consists of two main sections: the expanded waveguide combined with a taper that converts TM_0_ to TE_1_ using the mode hybridization principle, and an adiabatic directional coupler (ADC) that couples TE_1_ to TE_0_^2^. The corresponding optimized parameters were: $w_{H}=0.92 \mu m$, $l_{H}=74.5 \mu m$, $w_{ADC1}=1.25 \mu m$, $w_{ADC2}=0.466 \mu m$, $w_{g}=0.2 \mu m$ and $l_{\mathrm{ADC}}=10.25 \mu m$.

We aimed to verify the PRBS performance using the structure shown in Supplementary Figure S6e, where two mirrored PRBS structures are connected, enabling fiber-grating measurement on both sides with identical gratings designed for TE polarization. The results of the measurements from Port 2 to 3 indicated that the TM to TE conversion incurs a 6 dB loss over the spectrum, while the TE, as measured from Port 1 to 4, input to the FTS port experienced low loss, allowing accurate measurements. The TM to TE conversion loss, however, was significant enough to render this structure unsuitable for precise calibration. The loss from Port 1 to 3 indicated a loss of over 27 dB, hitting our measurement limit and indicating a high loss of over 21 dB for the TM polarization propagating straight through the PRBS. This result shows that the FTS is largely unaffected by the TM_0_ coupling into the chip as it is essentially filtered out. Therefore, to avoid confusion, we decided not to use the PRBS for calibration and to present the full structure in the main text. Additional simulations revealed that the ADC response has a very low tolerance to variations in slab thickness. As a result, any remaining TE_1_ mode in the main waveguide is filtered out after the PRBS tapering to the single mode condition, hinting at the reason for the high loss of TM mode propagating straight through the PRBS.

Supplementary Figures S6f and S6g illustrate the measurement setup of the VGC transmission and diffraction efficiency, simulation, and measurement results, respectively. A vertical multi-mode fiber (MMF) with a core diameter of 105 µm and $NA = 0.22$ was used to collect the diffracted light from the VGC. The transmission and diffraction efficiency varied between -4 dB to -6 dB of the input power after calibration with the response of the linear grating couplers. From simulations and the numerical model, we observed that the spectral modulation is due to reflections between the top-oxide-air interface and the VGC reflector, with relatively small changes in the top-oxide thickness causing significant shifts in the modulation.

To characterize the mirror reflection, we designed a structure, as shown in Supplementary Figure S6h, consisting of two directional couplers terminated by a mirror at one end. Maximum transmission through this structure is expected when the directional coupler is perfectly balanced, with any loss attributed to the mirror reflection and grating couplers. We measured multiple such structures with varying directional coupler lengths at an 860 nm wavelength, resulting in the structure’s reflection efficiency (with the square root corresponding to the mirror reflection at its maximum) shown in Supplementary Figure S6i. We observed a loss of approximately 1.6 dB and 1.9 dB from two different chips, higher than the expected 0.9 dB. The discrepancy can be attributed to the tilted mirror, as indicated by the SEM images, as well as the mirror’s surface roughness.

# S4. Spectrum Reconstruction

## Measurement Setups and Beam Characterization

In this section, we provide an overview of the setups used for T-matrix and Raman spectroscopy measurements, along with the characterization of the light sheet for T-matrix measurements, the 785 nm excitation spot on the sample, and the Raman signal light sheet. The operation of both setups is detailed in the Methods section, but here we elaborate on beam characterization in terms of full-width half-maximum (FWHM) width and length.

Supplementary Figure S7a shows the T-matrix characterization setup. Initially, we used an SMF, instead of MMF, connected to a single-mode tunable source, where L1 was an f = 60 mm lens. The resulting light sheet at the chip location had a width of 18.7 µm and a length of 1.5 mm, as shown in Supplementary Figure S7b. This light sheet was considerably shorter than the FTS and exhibited interference fringes. To avoid these issues in the T-matrix characterization, where any additional spectral response from outside the chip must be minimized, we used an integrating sphere and MMF input. Supplementary Figure S7c shows the improved light sheet used for T-matrix measurements, with a length of 2.9 mm and a width of 600 µm, demonstrating high uniformity.

Supplementary Figure S7e shows the 785 nm excitation spot characterized at the sample plane. The beam radius was measured to be 14.7 µm. Assuming that L1’s numerical aperture is fully filled by the Raman signal and the beam collection is limited only to the focal point, with no additional scattering that broadens the Raman signal, the optical throughput is calculated as $G=0.0007 \mathrm{mm}^{2}-sr$, which is seven times higher than the throughput of our FTS. However, as discussed in Supplementary Information S3 for an MMF, only 3% of the Raman signal from the excitation spot with a radius of 14.7 µm is collected due to the 1D array input configuration of our FTS. Indeed, this assumption is an oversimplification, as it assumes a non-scattering medium where Raman signal generation is confined to the area of the excitation laser spot, and a diffraction-limited light sheet perfectly matched to the edge coupler array.

We also characterized the light sheet resulting from the Raman spectrum of Paracetamol, as shown in Supplementary Figure S7f. No interference fringes were observed. The light sheet had a width of 210 µm and a length of 3.1 mm. Although the width is significantly larger than the edge coupler’s aperture, it provides a high tolerance for alignment and minimizes variation during measurements.


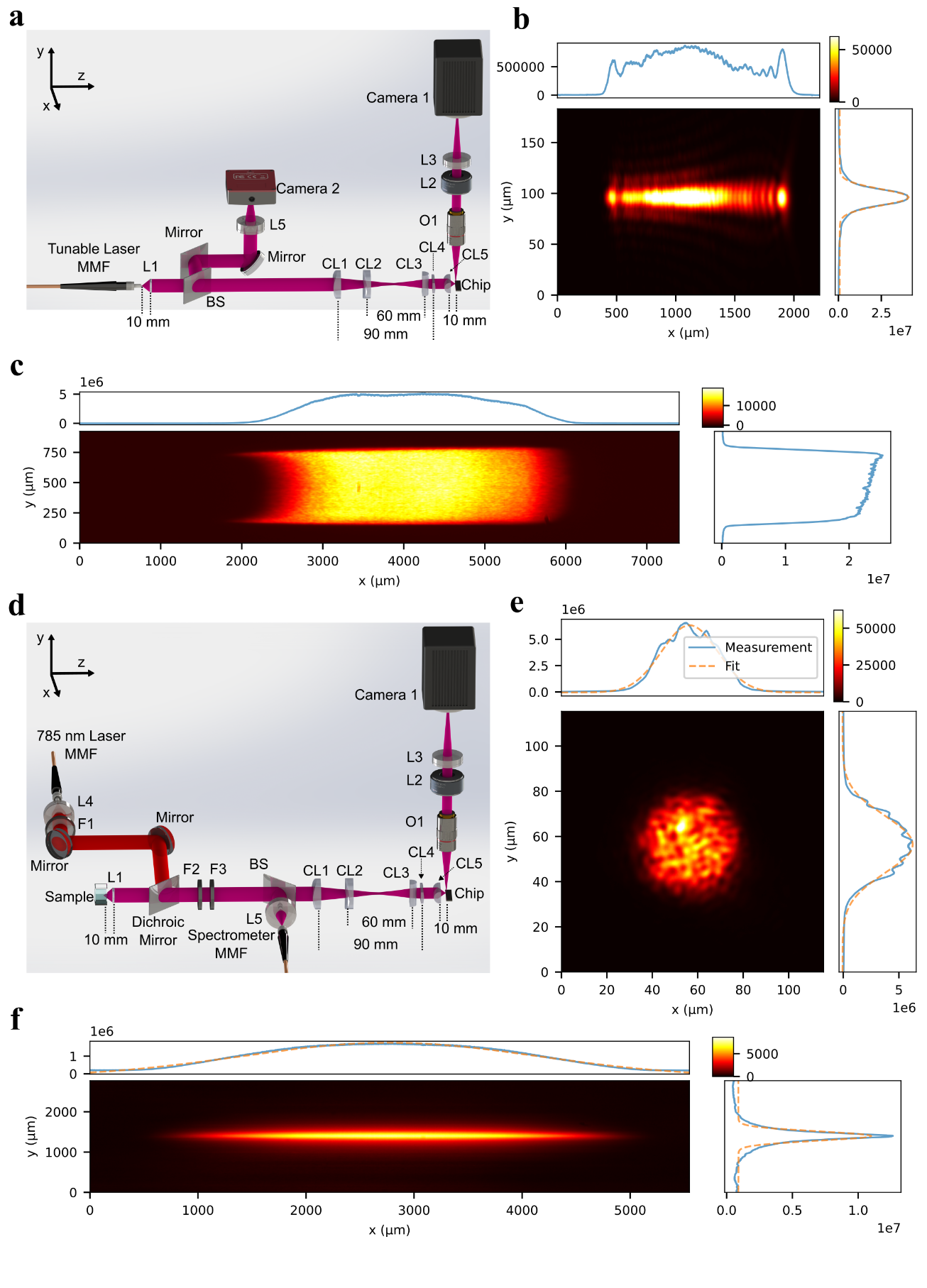


Supplementary Fig. S7| T-matrix measurement and Raman Spectrum reconstruction setups and beam characterization. **a**, T-matrix measurement setup. **b**, The light sheet profile at the chip plane from SMF input. **c**, The light sheet profile at the chip plane after using integrating sphere and MMF input. **d**, Raman spectrum reconstruction setup. **e**, 785 nm excitation beam profile at the sample plane. **f**, The light sheet profile of Paracetamol Raman signal at the chip plane.

| **Component** | **Vendor** | **Model Number** | **Specifications** |
| --- | --- | --- | --- |
| Tunable Laser | SUPERLUM | BS-840-1-HP | 20 mW power, 0.06 nm line width, 75 nm tuning range |
| 785 nm Laser | RealLight | NLMO-785-IFF-500 | 600 mW max power |
| Integrating Sphere | Labsphere | 3P-GPS-033-SL |  |
| Tunable Laser MMF |  | QP-I-600-0.55 | Core diameter 600 µm，NA = 0.55 |
| 785 nm Laser MMF |  |  | Core diameter 105 µm，NA = 0.22 |
| Spectrometer MMF |  |  | Core diameter 105 µm，NA = 0.22 |
| Spectrometer | Wasatch Photonics | WP-785XM-R0-S-OEM-50 | Sensor type: CCD, Pixel Size: 14 µm x 14 µm, Grating Type: Transmission, Slit size: 25 µm, f/1.3, NA: 0.38, Resolution: 13 cm^-1^ |
| Beam Splitter | Thorlabs | BS2R55-B | 50:50 |
| Dichroic Mirror | Semrock | Di02-R785-25×36 | 785 nm Long Pass |
| L1 | Thorlabs | AL1210-B | f = 10 mm, NA =0.55 |
| L2 | Thorlabs | TTL200-B | f = 200 mm |
| L3 | Thorlabs | AC254-150-B | f = 150 mm |
| L4 | Thorlabs | AC254-030-B | f = 30 mm |
| L5 | Thorlabs | AC254-040-B | f = 40 mm |
| CL1 | LBTEK | CY107R12 | f_y_ = 50 mm |
| CL2 | LBTEK | CY107R12 | f_x_ = 50 mm |
| CL3 | LBTEK | CY104206-B | f_x_ = 10 mm |
| CL4 | LBTEK | CY104211-B | f_y_ = 40 mm |
| CL5 | LBTEK | CY104206-B | f_y_ = 10 mm |
| F1 | Semrock | LL01-785-25 | 785 nm Laser line, OD>5 |
| F2 | Semrock | LP02-785RU-25 | 785 nm Long pass, OD>6 |
| F3 | Thorlabs | FBH850-40 | 850 nm Band pass, FWHM = 40 nm |
| O1 | Thorlabs | MY5X-822 | NA = 0.14 |
| Camera 1 | Hamamatsu | C13440-20CU |  |
| Camera 2 | Thorlabs | CS165CU/M |  |

### Supplementary Table 1| List of components used for T-matrix measurement and Raman spectrum reconstruction, as illustrated in Supplementary Figures S7a and S7d.

## Interferometer Array Cavity Response in the Raman Spectrum Reconstruction Measurements

**
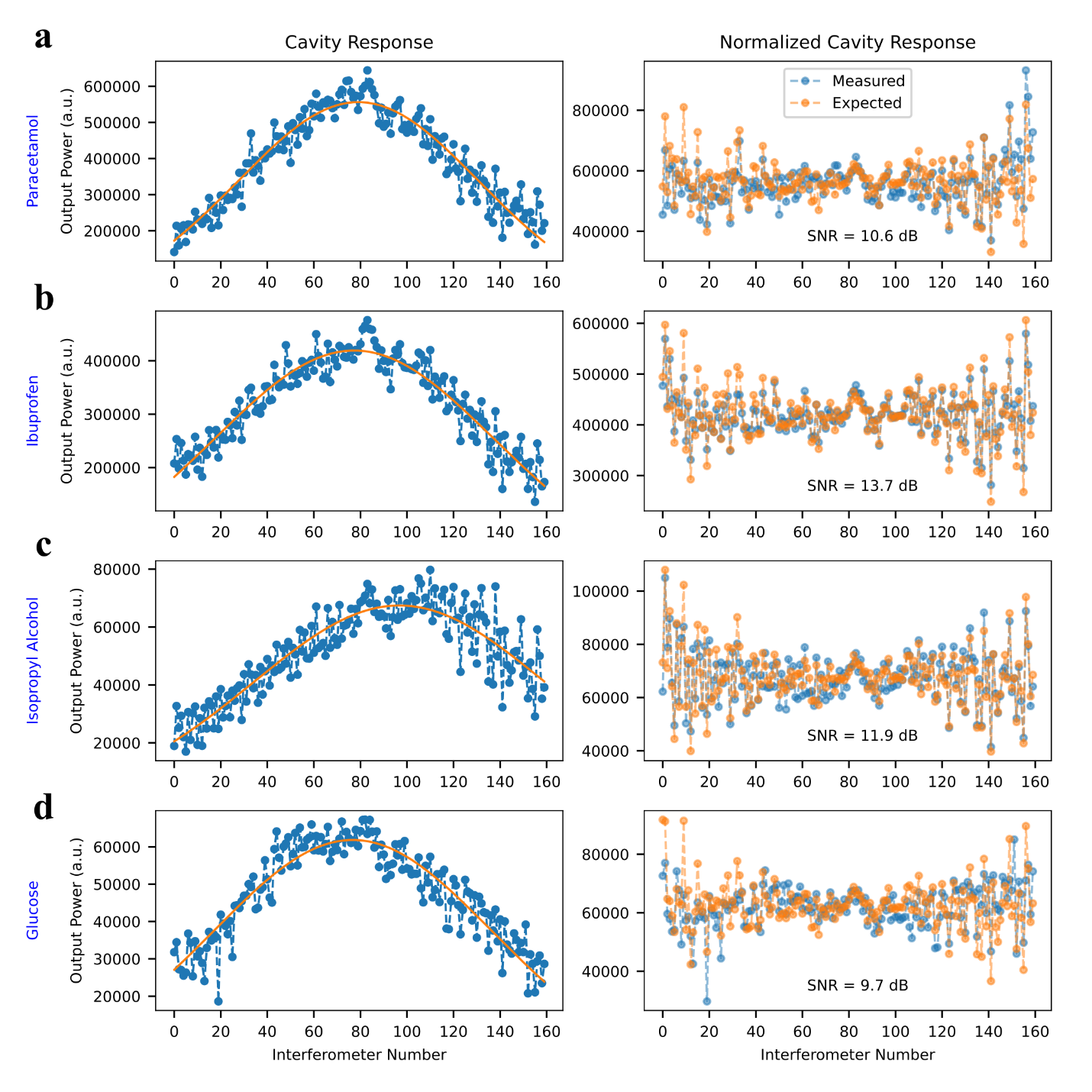
**

Supplementary Fig. S8| The Raman spectrum interferometer responses for different cavity lengths in the FTS. **a**, Paracetamol cavity responses before and after light sheet normalization. **b**, Ibuprofen cavity responses before and after light sheet normalization. **c**, Isopropyl Alcohol cavity responses before and after light sheet normalization. **d**, Glucose cavity responses before and after light sheet normalization.

The first step in reconstructing the Raman spectrum involves recording the interferometer responses at varying cavity lengths in the FTS. The focused light sheet from the Raman signal was aligned to the edge of the chip, and interferometer responses were recorded for Paracetamol, Ibuprofen, isopropyl alcohol, and glucose. Supplementary Figure S8 displays the interferometer responses both before (left) and after normalization (right) of the Gaussian light sheet profile. The light sheet profile was determined through fitting and is indicated in the graphs alongside the cavity response before normalization.

After normalization and multiplication with the maximum value of the fit, we compared the data with the expected interferometer response of each substance, which was calculated as outlined earlier in this section. To quantify the SNR, we took the median of the variation of each measured point from the expected response.

We found that the trend of the measured values aligned well with the expected response, with all substances exhibiting an SNR of around 10 dB or higher. However, this is still below the 15 dB detection limit required for the Ibuprofen spectrum under Gaussian noise. The measured response exhibited smooth deviations from the expected response, especially for the shortest and longest interferometers, which we attribute to the normalization process of the light sheet. While these deviations reduce the overall SNR, their impact on the reconstructed spectrum is likely less significant compared to random noise of Poisson or Gaussian nature.

Among the samples, Paracetamol showed the highest peak intensity, while glucose displayed the lowest. Due to its low intensity combined and spectral richness, we expect glucose to exhibit the poorest reconstructed spectrum, while Paracetamol is expected to yield the best results in terms of matching the reconstructed spectrum.

There were significant variations in the output power responses for the interferometers with longer cavity lengths, even for spectrally rich spectra such as Ibuprofen. This was unexpected based on our initial numerical analysis presented in Figure S1. These variations suggest the presence of a slowly varying, wavelength-dependent response, potentially arising from reflections between the top-oxide-air interfaces and VGC reflectors.

## Influence of Signal-to-Noise Ratio (SNR) on Spectrum Reconstruction for Different Regression Methods

We aimed to develop a numerical understanding of how different regression methods perform under varying SNR conditions of the cavity response. Specifically, we studied the Penrose-Moore pseudoinverse, LASSO, Ridge, and Elastic Net regressions for the reconstruction of the Ibuprofen signal, which exhibits a relatively rich spectrum.

Firstly, the Raman spectrum of Ibuprofen from a conventional spectrometer was obtained (denoted as $x$). Using the noise-free T-matrix from our numerical model, which represents the response of our FTS (denoted as $A$), we calculated the cavity response ($y$) through matrix multiplication $Ax=y$.

Next, Gaussian noise was added to the cavity response using a random number generator from Numpy, resulting in a noisy signal $\hat{y}=y+\varepsilon$, where $\sigma_{\varepsilon}=\frac{y}{SNR}$ for each element in the vector $y$.

We then solved the equation $A\hat{.x}=\hat{y}$ for varying SNR by optimizing the hyperparameters, as well as the Savitzky-Golay window length and polynomial order. To quantify the performance, we calculated the Pearson Correlation Coefficient. The random number generation and regression process were repeated five times, and the Pearson Correlation Coefficient for each SNR and regression method was averaged.

Supplementary Figures S9a, S9b, and S9c illustrate the reconstructed spectrum at various SNR levels (3 dB to 30 dB), the Pearson Correlation Coefficient ($r$) of the solutions, and the corresponding hyperparameters for each SNR. We observed that the pseudoinverse method required approximately 18 dB SNR for effective reconstruction, while all other regression methods achieved satisfactory performance at 15 dB. Additionally, the pseudoinverse method’s reconstruction was capped at an $r$ value of 0.8, whereas all other methods performed nearly equally well, surpassing $r=0.9$. In terms of $r$ value, no significant differences were observed among the regression methods except for the pseudoinverse.

The hyperparameter variation shown in Supplementary Figure S9c indicates that Ridge regression converges at 17 dB SNR, while LASSO regression stabilizes after 19 dB SNR, aligning well with the 15 dB SNR detection limit. Elastic Net regression, however, displayed fluctuations in its L1-norm hyperparameter between two values, indicating difficulties in balancing sparsity.

The spectra shown in Supplementary Figure S9a reveal that the pseudoinverse method retains the background, whereas it is effectively suppressed by the other regression methods. Around the 15 dB SNR detection limit, LASSO regression tends to miss minor peaks due to its sparse nature, while Ridge regression introduces artificial peaks to smooth the spectrum. Elastic Net regression maintains a smooth spectrum without generating fake peaks, albeit requiring the optimization of two hyperparameters. As our objective is to minimize optimization while avoiding artificial peaks in the reconstructed spectrum for efficient material identification, we focused on LASSO regression combined with Savitzky-Golay smoothing within the scope of this article.

**
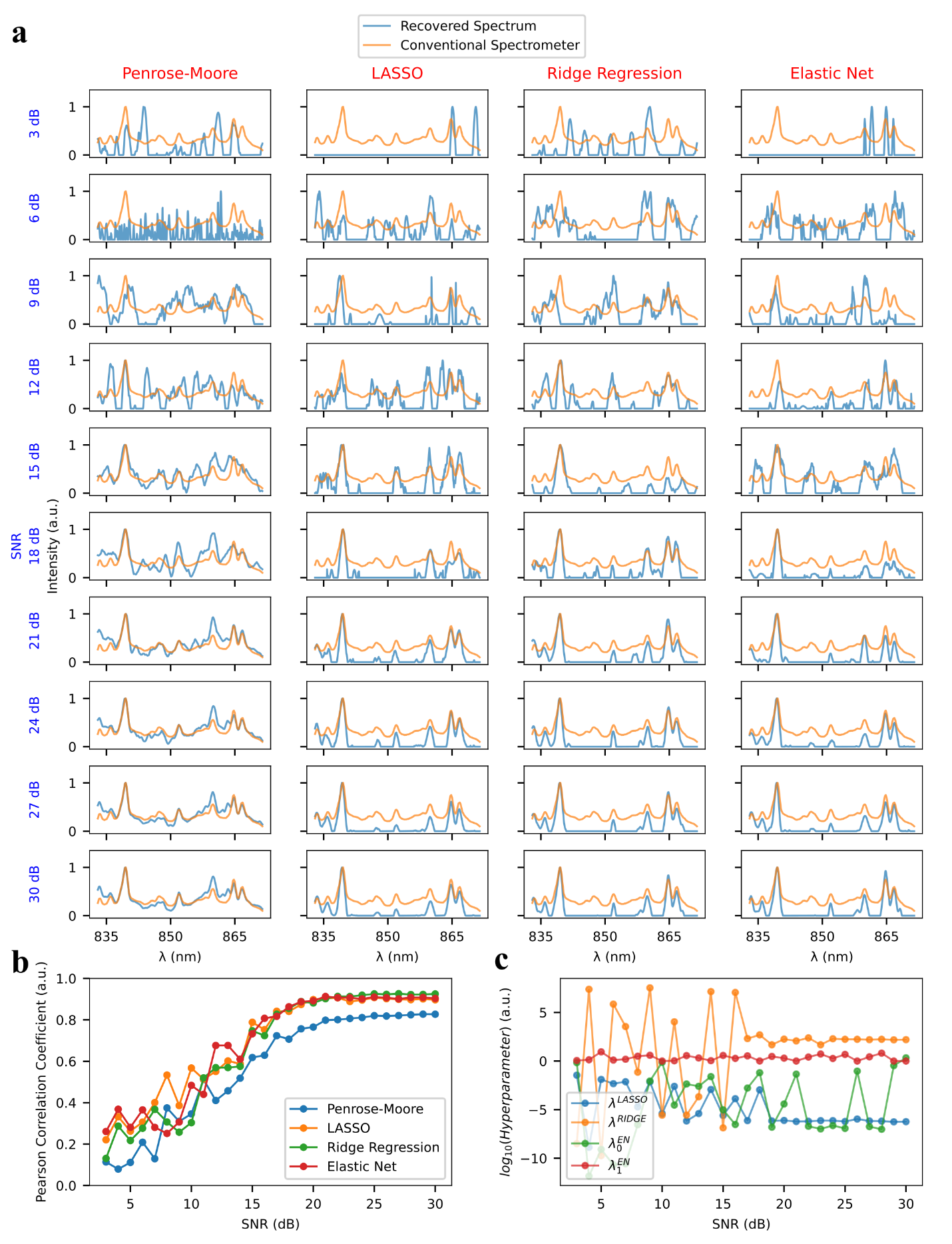
**

Supplementary Fig. S9| Spectrum reconstruction using different regression methods with added noise to the cavity response. **a**, The reconstructed spectrum using the Penrose-Moore pseudoinverse method, LASSO, Ridge, and Elastic Net regressions for varying SNR. **b**, Averaged Pearson Correlation Coefficient of the reconstructed spectrum for varying SNR levels. **c**, Optimized hyperparameters for the different regression methods at varying SNR levels.

## Measured Raman Spectrum Reconstruction Dependence On The Regression Method

We evaluated the Raman spectrum reconstruction using four regression methods: (1) Penrose-Moore pseudoinverse, (2) LASSO, (3) Ridge, and (4) Elastic Net regression. Based on the measured T-matrix after light-sheet normalization, we reconstructed the Raman spectra for all four substances, as shown in Supplementary Figure S10. The Pearson Correlation Coefficients and hyperparameters are indicated for each regression method and substance.

Elastic Net and LASSO regression, when combined with Savitzky-Golay smoothing (despite some peak shifts), outperformed the other methods for all substances. The Penrose-Moore pseudoinverse produced viable spectra only for Paracetamol and IPA, where the signal was either high or the spectrum was relatively simple. Ridge regression, which was expected to perform comparably to Elastic Net and LASSO regression, resulted in a poorer spectral match, particularly for Ibuprofen and glucose, due to the generation of artificial peaks that degraded the Pearson correlation coefficient. This issue likely arose because the SNR for these substances was slightly below their detection threshold.

For all regression methods, the reconstructed spectra for Paracetamol and IPA showed good agreement with the expected results, indicating that their SNR exceeded the detection threshold. There are two possible reasons for this. First, the spectral richness of Paracetamol and IPA is lower than that of Ibuprofen, which reduces the required detection SNR. Second, the smooth variations in the interferometer response for Paracetamol and IPA may have had less impact on the spectrum reconstruction than random noise fluctuations, which are more problematic for substances with rich spectral features like Ibuprofen and glucose.


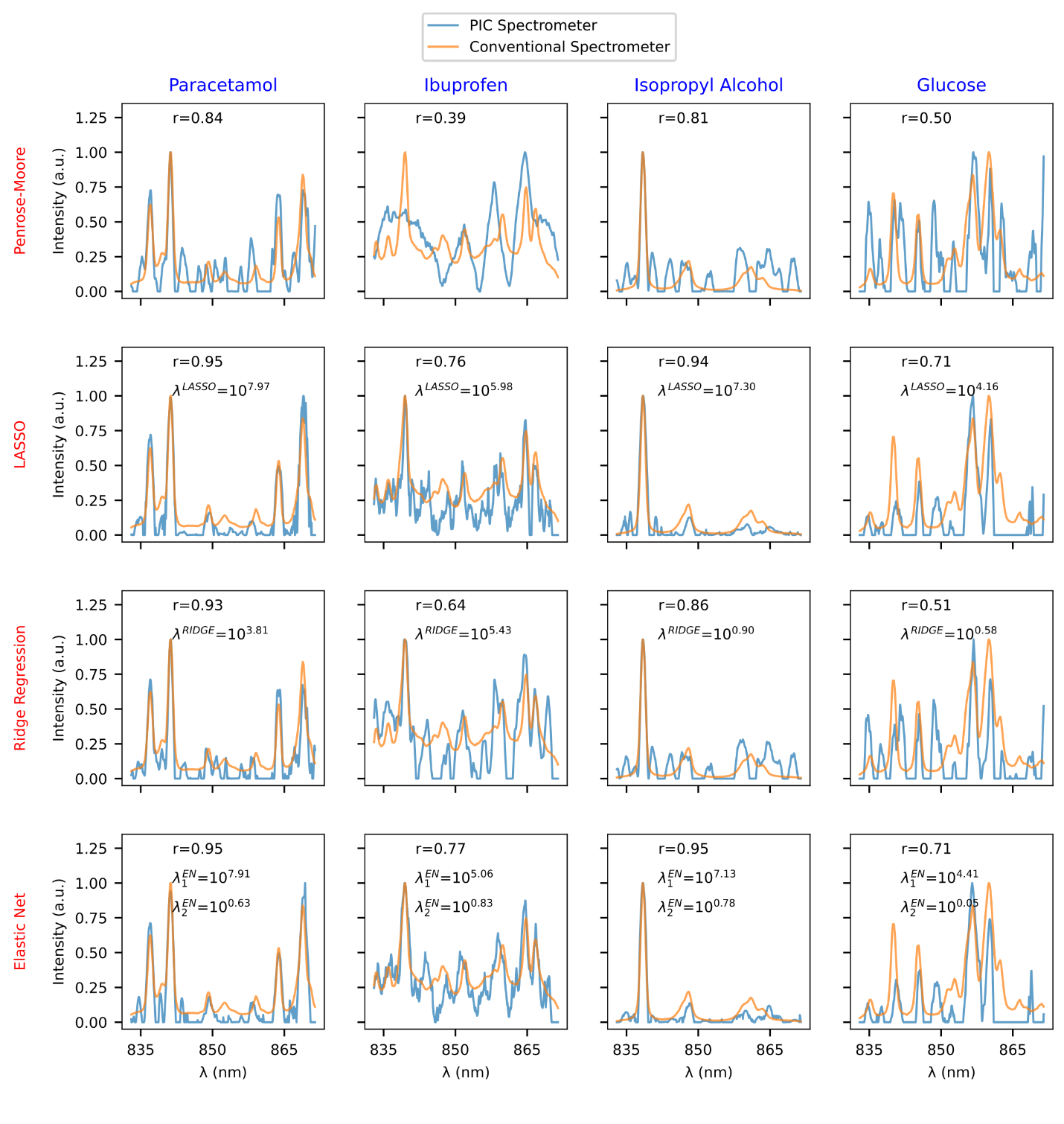


Supplementary Fig. S10| Raman spectra of four substances reconstructed from the measurements using different regression methods.

## Measured Raman Spectrum Reconstruction Dependence On The Number of Images

We analyzed the minimum number of images (or signal strength) required for the spectrum reconstruction of the four substances. Starting with 1 image and increasing the number of images up to 64 images, with each step summing 4-fold (equivalent to a 3 dB increase in SNR, assuming shot-noise limited measurements), we used LASSO regression combined with Savitzky-Golay smoothing to reconstruct the spectra. This analysis also provides insight into our proximity to the detection SNR threshold. The reconstructed spectra from the measurements are shown in Supplementary Figure S11. Paracetamol and IPA were successfully reconstructed with a single image, showing little improvement with additional signal and indicating that even a single image exceeds their corresponding detection SNR threshold. In contrast, Ibuprofen and glucose, both exhibiting a complex spectral richness, showed progressively better reconstruction with an increasing number of images, showing that more signal is needed for optimal spectrum reconstruction.


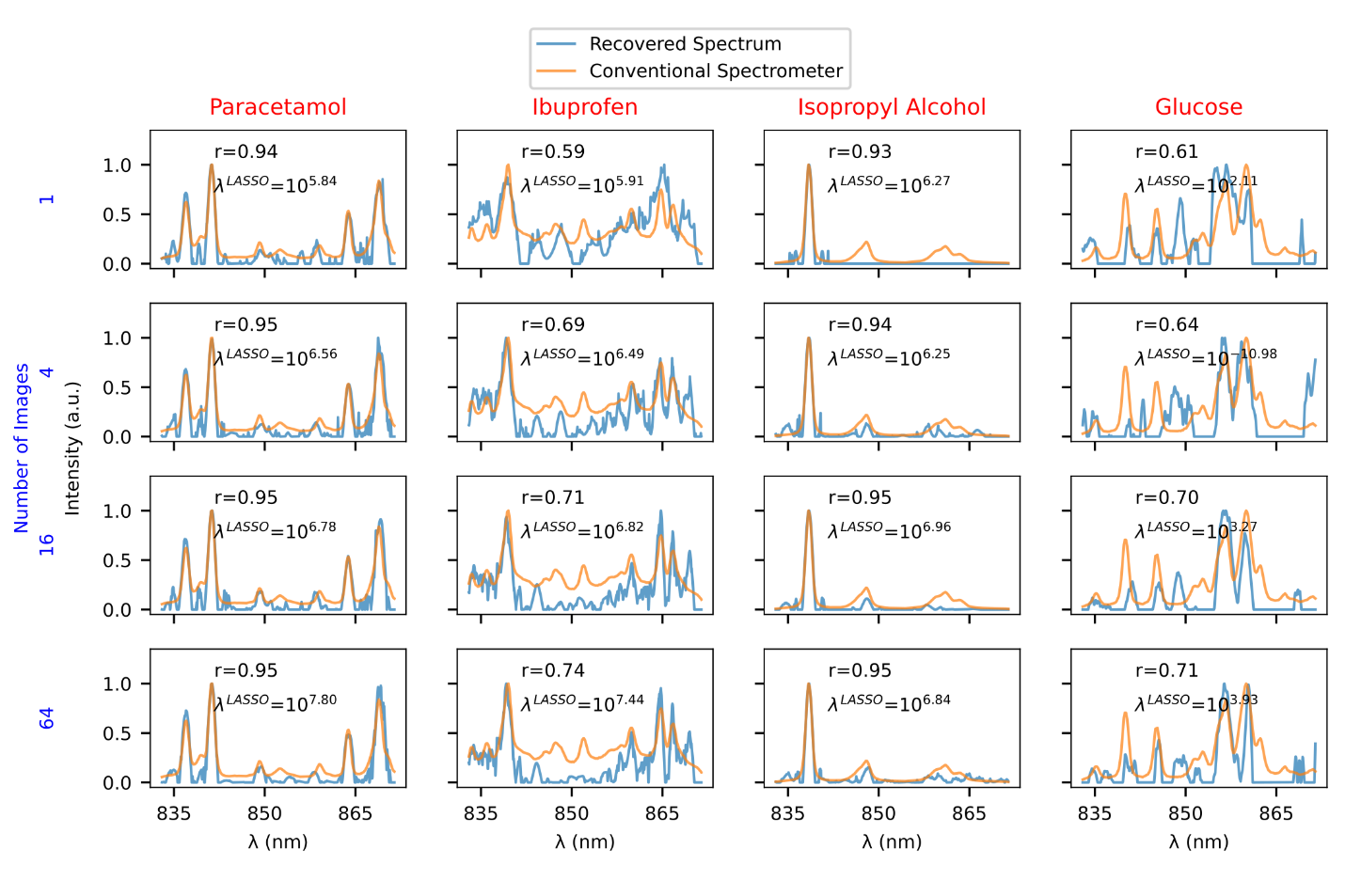


### Supplementary Fig. S11| Raman spectra of four substances reconstructed from the measurements using varying numbers of images and LASSO regression.

## Measured Raman Spectrum Reconstruction Using LASSO Regression With Varying Hyperparameter Value


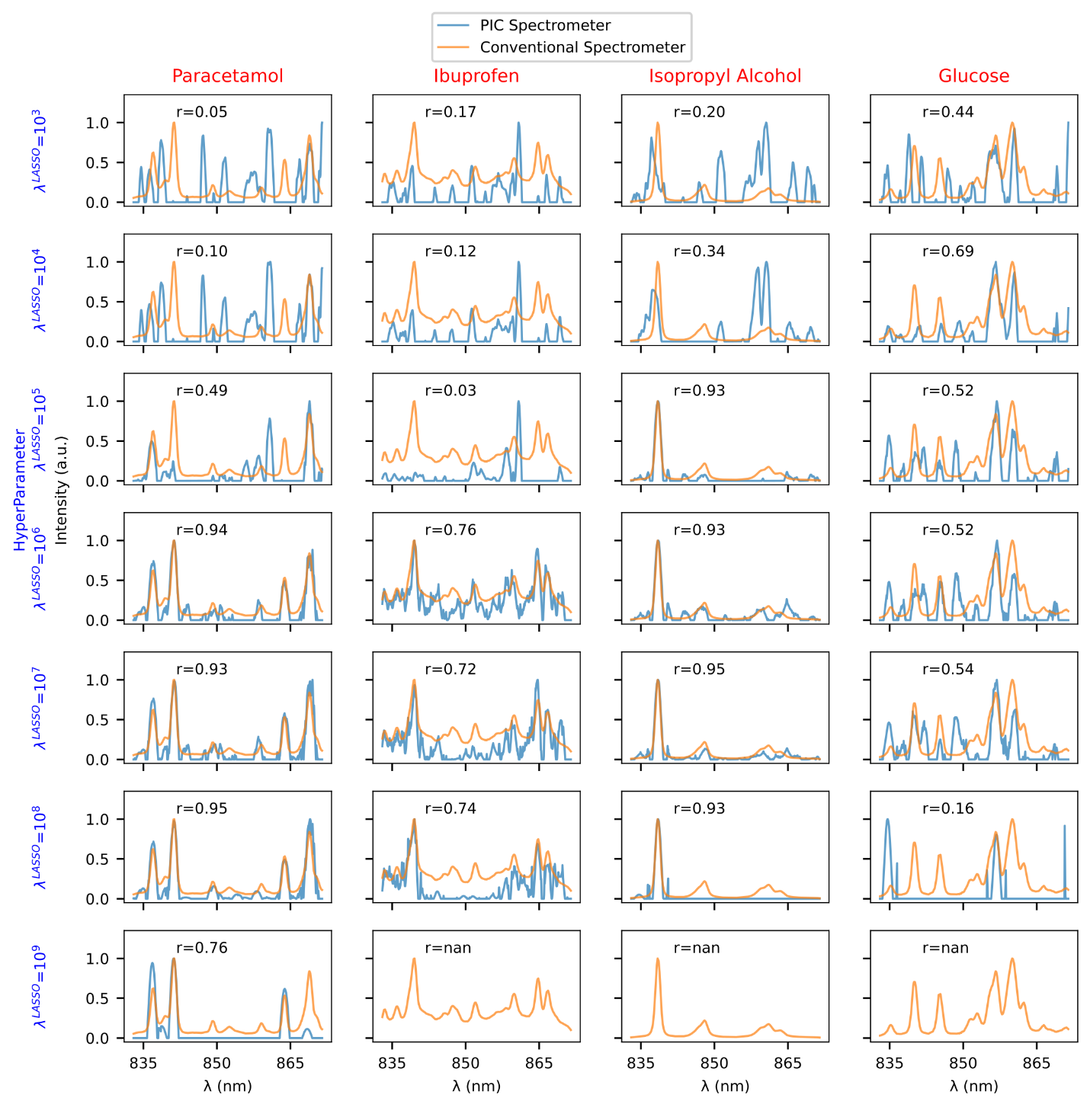


### Supplementary Fig. S12| Raman spectra of four substances reconstructed from the measurements using varying LASSO regression hyperparameter values.

The Raman spectra of the four substances were reconstructed using LASSO regression with varying hyperparameter values. We observed that selecting a single nearly optimal hyperparameter for all substances could be possible. The reconstructed spectra, along with their corresponding Pearson correlation coefficients, are shown in Supplementary Figure S12. After performing the LASSO regression for each substance, we applied a Savitzky-Golay smoothing with polynomial order and window length values from the optimized solutions (presented in Supplementary Figure S10) to maintain consistency.

We found that each substance had a different optimal hyperparameter value, with a relatively wide range yielding satisfactory results. Higher values of the hyperparameter led to the disappearance of peaks due to sparsity, while lower values generated artificial peaks. Despite variations in signal levels and spectral richness among the substances, $\lambda^{\mathrm{LASSO}}={10}^{6}$ provided a nearly optimum solution for all substances except glucose. We believe that glucose is an exception due to its low signal level and spectral richness, which results in an SNR that is considerably below its detection threshold compared to the other substances.

# S5. Concentration Detection From The Reconstructed Spectra

We demonstrated the detection of IPA concentration in a water solution using the reconstructed spectrum from the FTS. LASSO regression was employed to reconstruct all spectra. Supplementary Figure S13 presents the reconstructed spectra for each concentration with optimization (a), using the same hyperparameter value and smoothing parameters for all reconstructions (b), and the corresponding peak amplitude for varying IPA concentrations (c and d). The percentages shown are in percentage volume.

We observed that a well-matching spectrum can be reconstructed down to a 5% IPA concentration. The peak amplitudes of the reconstructed spectra were then analyzed and normalized to the peak amplitude from the highest concentration, and compared with the peak amplitudes from a conventional spectrometer. When each concentration was optimized with different hyperparameters and smoothing values, the peak amplitudes showed significant deviation from the expected values (Supplementary Figure S13c). To address this, we used the same parameter set for reconstructing all spectra at varying concentrations (Supplementary Figure S13b) and reanalyzed the peak amplitudes, as illustrated in Supplementary Figure S13d.

We found that the measured normalized peak amplitudes closely matched the expected values, with about a 10% error, down to a 25% IPA concentration. Below this concentration, the peak amplitude remained proportional to the IPA concentration, suggesting that accurate concentration measurement could be achieved after calibration. However, this would require multiple measurements at different concentrations to validate the results. In conclusion, precise concentration detection requires further investigation, potentially requiring improved SNR and higher optical throughput.


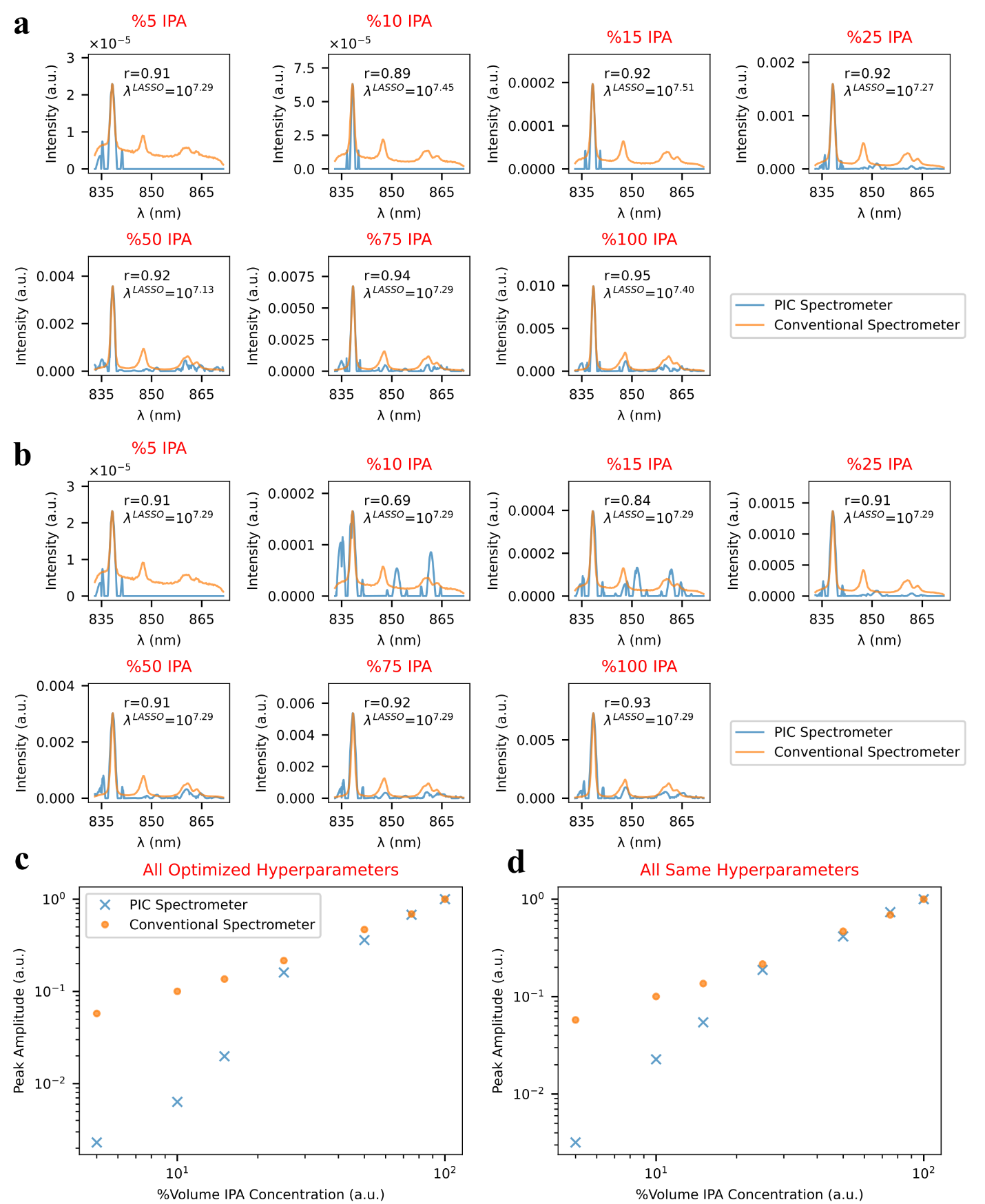


Supplementary Fig. S13| Concentration detection of IPA in water solution using the peak amplitude of its Raman spectra reconstructed from the FTS. **a**, Reconstructed spectra after optimization for each concentration. **b**, Reconstructed spectra using the same parameter set for all concentrations. **c**, Normalized peak amplitudes from the optimized reconstructed spectra for varying concentrations. **d**, Peak amplitudes from the reconstructed spectra when a single parameter set was applied to all regressions.

# S6. Total Efficiency of the Waveguide-Based FTS Raman Spectroscopy Setup

In this section, we give an overview of the total efficiency of the Raman spectroscopy setup, including the waveguide-based FTS, and demonstrate that approximately 0.21% of the Raman signal from the Paracetamol sample reaches the camera. The primary source of loss is the mismatch between the optical throughput of the Raman signal and the FTS.

Supplementary Table 2 summarizes the efficiency of each segment of propagation from the substance to the camera. The transmission efficiencies of the free-space components were obtained from the specification sheets provided by the vendors, and the total free-space component loss is calculated to be nearly 3 dB.

The losses originated from the FTS, approximately 24 dB, and were divided into two parts: coupling into the FTS, and propagation and outcoupling from the FTS. We assumed that the mode is uniformly confined within the waveguides at the edge coupler facet, resulting in a fill factor penalty of 75%.

An optical throughput mismatch penalty of 19.3 dB was calculated based on the Paracetamol Raman signal image shown in Supplementary Figure S7f. We observed that the light-sheet length matched the length of the interferometer array without any focusing in the optical system. Thus, we attributed the majority of this penalty to the light-sheet width. Using the $NA=0.22$ of the light-sheet focusing and a width of 210 µm, we calculated the throughput mismatch penalty as previously detailed in Supplementary Information S2.

The loss from the FTS, 6.22 dB, was calculated using the measured single-mode waveguide propagation loss, 1.18 dB cm^-1^, the loss of TM polarization (assuming half of the light is TM polarized), and the average simulated out-coupling efficiency of the interferometers (Supplementary Figure S5). The waveguide width of the majority of the propagation is wider than that of a single-mode waveguide, making the propagation loss smaller than 1.18 dB cm^-1^ over ~1 mm propagation in the waveguides.

We estimate that the FTS Raman setup would exhibit a similar free-space propagation loss compared to that of a diffraction-type spectrometer, with the primary difference being optical throughput. As a result, the efficiency of this system for Paracetamol Raman spectroscopy would be approximately $5.6\%\times24\%=1.3\%$ that of a diffraction-type spectrometer with a slit width of 25 µm and f/1.3.

| **Part** | **Component** | **Transmission (%)** | **Loss (dB)** |
| --- | --- | --- | --- |
| Sample to FTS Propagation | L1 | 98 | 0.09 |
|  | Dichroic Mirror | 95 | 0.22 |
|  | F2 | 96 | 0.18 |
|  | F3 | 95 | 0.22 |
|  | CL1 | 98 | 0.09 |
|  | CL2 | 98 | 0.09 |
|  | CL3 | 98 | 0.09 |
|  | CL4 | 98 | 0.09 |
|  | CL5 | 98 | 0.09 |
| Sample to FTS Propagation Total | | 77 | 1.16 |
| FTS Coupling | Fill Factor Penalty | 75 | 1.25 |
|  | Optical Throughput Penalty | 1.17 | 19.31 |
| FTS Coupling Total | | 0.88 | 20.56 |
| FTS | Waveguide Loss | 97 | 0.12 |
|  | Polarization Penalty | 50 | 3.00 |
|  | Interferometer Loss | 49 | 3.10 |
| FTS Total | | 24 | 6.22 |
| FTS to Camera | O1 | 70 | 1.55 |
|  | L2 | 98 | 0.09 |
|  | L3 | 96 | 0.18 |
| FTS to Camera Total | | 66 | 1.82 |
| Raman Spectroscopy Setup Total | | 0.11 | 29.76 |

### Supplementary Table 2| The breakdown of the losses of waveguide-based FTS Raman setup.

# Supplementary References

1. Nikkhah, V. *et al.* Inverse-designed low-index-contrast structures on silicon photonics platform for vector-matrix multiplication. *Nat. Photon.* **18**, 501–508 (2024).

2. Xu, H. & Shi, Y. Ultra-broadband silicon polarization splitter-rotator based on the multi-mode waveguide. *Opt. Express* **25**, 18485 (2017).

3. Schiappelli, F. Efficient fiber-to-waveguide coupling by a lens on the end of the optical fiber fabricated by focused ion beam milling. *Microelectronic Engineering* **73–74**, 397–404 (2004).

4. Hodara, H. & Slemon, C. Throughput and coupling in optical fibers [J]. *Applied Scientific Research* **41**, 203-224 (1984).

5. Van Dorpe, P. & Peumans, P. Optical spectrometer with matched étendue. U.S. Patent 9,909,992[P]. 2018-3-6.

6. Raman Spectrometers & Systems. *Wasatch Photonics* https://wasatchphotonics.com/product-category/spectrometers/raman-spectrometer-solutions/.
